# Prognostic Value of CXCR2 in Breast Cancer

**DOI:** 10.3390/cancers12082076

**Published:** 2020-07-27

**Authors:** Florence Boissière-Michot, William Jacot, Julien Fraisse, Sophie Gourgou, Colin Timaxian, Gwendal Lazennec

**Affiliations:** 1 Institut Régional du Cancer de Montpellier (ICM), Val d’Aurelle, 34298 Montpellier, France; Florence.Boissiere@icm.unicancer.fr (F.B.-M.); William.Jacot@icm.unicancer.fr (W.J.); Julien.Fraisse@icm.unicancer.fr (J.F.); Sophie.Gourgou@icm.unicancer.fr (S.G.); 2 Faculty of Medicine, Montpellier University, INSERM U1194, 34090 Montpellier, France; 3 Centre National de la Recherche Scientifique (CNRS), SYS2DIAG-ALCEDIAG, Cap delta, 1682 Rue de la Valsière, 34184 Montpellier, France; timax.co@gmail.com; 4 Centre National de la Recherche Scientifique (CNRS), Groupement de Recherche (GDR) 3697 “Microenvironment of Tumor Niches”, Micronit, France

**Keywords:** breast cancer, chemokines, cytokines, CXCR2, neutrophils, macrophages

## Abstract

The tumor microenvironment appears essential in cancer progression and chemokines are mediators of the communication between cancer cells and stromal cells. We have previously shown that the ligands of the chemokine receptor CXCR2 were expressed at higher levels in triple-negative breast cancers (TNBC). Our hypothesis was that CXCR2 expression could also be altered in breast cancer. Here, we have analyzed the potential role of CXCR2 in breast cancer in a retrospective cohort of 105 breast cancer patients. Expression of CXCR2, CD11b (a marker of granulocytes) and CD66b (a marker of neutrophils) was analyzed by immunohistochemistry on tumor samples. We demonstrated that CXCR2 stained mainly stromal cells and in particular neutrophils. CXCR2, CD11b and CD66b expression were correlated with high grade breast cancers. Moreover, TNBC displayed a higher expression of CXCR2, CD11b and CD66b than hormone receptor positive or Her2 positive tumors. High levels of CXCR2 and CD11b, but not CD66b, were associated with a higher infiltration of T lymphocytes and B lymphocytes. We also observed a correlation between CXCR2 and AP-1 activity. In univariate analyses, CXCR2, but not CD11b or CD66b, was associated with a lower risk of relapse; CXCR2 remained significant in multivariate analysis. Our data suggest that CXCR2 is a stromal marker of TNBC. However, higher levels of CXCR2 predicted a lower risk of relapse.

## 1. Introduction

The implication of the tumor microenvironment has gained growing interest from the past years and it is now well admitted that targeting tumor cells only could be insufficient to achieve optimal therapeutic responses. Tumor cells are surrounded by a stromal compartment including different types of extracellular matrix, signaling molecules and stromal cells, among which immune cells, fibroblasts, mesenchymal stem cells and endothelial cells will be found [1,2]. A variety of immune cells can be involved, including in particular, T lymphocytes, B lymphocytes, dendritic cells, macrophages, neutrophils and natural killers. These cells have the ability to establish contacts as well as distant interactions through the release of soluble factors. In turn, this will either favor or impair tumor growth, invasion and metastasis. One must emphasize that tumor cells are able to shape the stromal compartment though their physical or distant interactions with stromal cells, leading for instance to the acquisition of cancer associated fibroblast phenotype for fibroblasts or mesenchymal stem cells [3,4]. In a similar manner, recruitment of immune cells will also be affected by tumor cells and these cells may display pro- or anti-tumor properties. This has led to the identification of different types of tumor associated macrophages [5], as well as of tumor associated neutrophils [6,7].

Among the soluble factors secreted both by epithelial tumor cells and stromal cells, chemokines have been identified as key players. The superfamily of chemokines can modulate basically all events of tumor progression including growth, survival, angiogenesis, invasion, extravasation and metastasis [8,9]. Chemokines are chemotactic cytokines and have been divided in four subtypes (CXC, CC, C or CX3C), based on the location of cysteines in N-terminus of the protein [10]. They act through Gαi protein-coupled receptors leading to the activation of phosphatidylinositol-3 kinase/Akt, phospholipase C/protein kinase C and mitogen-activated protein kinase/p38, Ras/Erk and Janus kinase 2/signal transducer and activator of transcription (STAT3) and NF-κB pathways [11].

We have previously shown that the ligands of human chemokine receptor CXCR2 (CXCL1, CXCl2, CXCL3, CXCL5, CXCL6, CXCL7 and CXCL8) were coregulated in breast cancers, presumably because of their common location in a narrow region of chromosome 4q [12,13]. CXCR2 ligands are expressed at higher levels in triple negative breast cancer (TNBC), compared to luminal and Her2 breast tumors or cells lines [12,13,14]. Coexpression of CXCR2 ligands involves in particular nuclear factor-kappa B (NF-KB) and activator protein 1 (AP-1) pathways [12,15,16].

CXCR2, which is expressed at high levels in neutrophils and at lower levels in endothelial cells, appears essential in the control of angiogenesis, through the binding of tripeptide glutamic acid-leucine-arginine (ELR)-motif containing chemokines, present in the N-terminal part of the protein [17]. We hypothesized that CXCR2 expression could be also altered in breast cancer and could account for a differential recruitment in the tumor microenvironment.

The goal of this study was now to determine the expression and prognosis value of CXCR2 in breast cancer, which has been poorly studied.

## 2. Results

### 2.1. Validation of the Detection of CXCR2 Expression

Since our study has mainly evaluated CXCR2 expression, we attached particular importance to providing trustworthy CXCR2 immunostaining results. Based on previous publications and compatibility of the antibodies (Ab) for the immunohistochemistry (IHC) procedure, seven CXCR2 Ab have been selected and tested (Table S1).

We were unable to get any staining with the rabbit polyclonal LS-A804 and the mouse monoclonal 5E8-C7-F10 Ab, whatever the immunohistochemistry (IHC) procedure used. Two other monoclonal Ab displayed divergent patterns of staining, such as nuclear staining of epithelial cells (clone #48-311), or staining of endothelial cells and fibroblasts (clone 6D499; data not shown). Finally, three Ab (Clone E-2, # HPA031999 and clone 19) displayed, with various staining intensities and background, similar pattern of staining, i.e., scattered cells within the stroma (Figure S1A–C) or within blood vessels (Figure S1D–F).

**Figure 1:**
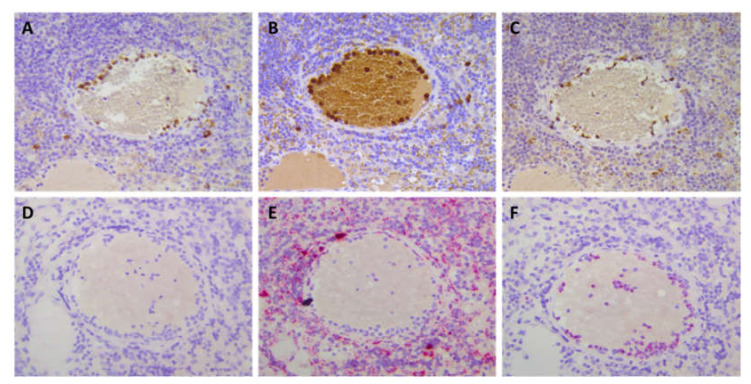
Validation of CXCR2 immunostaining with RNA in situ hybridization (ISH). Serial sections showing consistent staining of CXCR2 with the 3 Ab tested ((**A**): clone E-2; (**B**): rabbit polyclonal Ab HPA03199 and (**C**): clone 19) were submitted to ISH using a negative control probe (**D**) a positive HsPPIB control probe (**E**) and a specific HsCXCR2 probe. (**F**) Note a consistent pattern between immunohistochemistry (IHC; **A**–**C**) and ISH CXCR2 signals (**F**). Magnification 400×.

The specificity of CXCR2 immunostaining was thoroughly evaluated by staining serial sections using in situ hybridization (ISH). We showed a CXCR2 mRNA expression pattern consistent with CXCR2 protein expression as detected with clone E-2, #HPA031999 or clone 19 Ab (Figure 1). Due to its absence of background staining, the clone E-2 was selected for further IHC analyses. CXCR2 staining showed that stromal cells with a granulocytic shape, likely neutrophils, were accounting for the expression of the receptor. On the other hand, we could not detect an expression of CXCR2 in mammary epithelial cancer cells.

**Figure 2:**
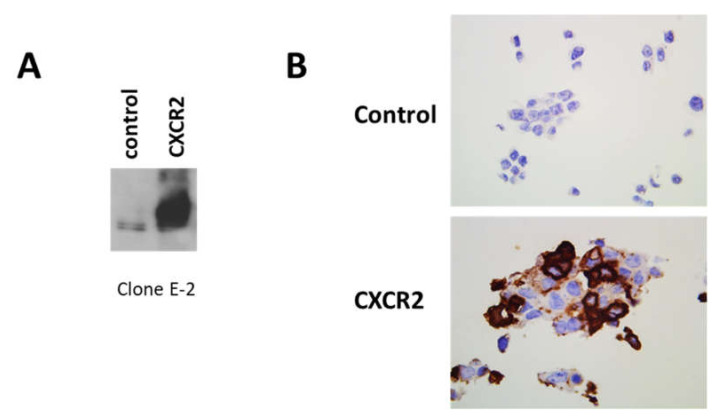
Validation of clone E-2 on HEK-293 cells transfected with human CXCR2 cDNA. (**A**) Proteins, extracted from HEK-293 cells mock transfected (left lane) or transfected with human CXCR2 cDNA (right lane), were analyzed by Western blot. (**B**) HEK-293 cells mock transfected (upper panel) or transfected with human CXCR2 cDNA (lower panel) and then hybridized in the same conditions as breast cancer tissues with clone E-2 Ab, but at a dilution of 1/5000. Magnification 630×.

We performed additional controls of specificity for clone E-2 CXCR2 Ab. First, using irrelevant IgG1 at the same concentration than the one used for E-2 CXCR2 Ab, we failed to detect any staining (Figure S2). Then, in Western blot experiments using proteins extracted from HEK-293 cells transfected or not with human CXCR2 cDNA, we observed that clone E-2 CXCR2 Ab could clearly detect a strong expression of CXCR2 in CXCR2-transfected cells (Figure 2A, Figure S3). Moreover, IHC experiments on a pellet of the same cells showed also a clear increased expression in CXCR2 transfected cells (Figure 2B), confirming the specificity of this Ab.

### 2.2. Analysis of CXCR2, CD66b and CD11b with Clinical Parameters of Breast Tumors

We analyzed by IHC CXCR2, CD11b and CD66b expression on a cohort of 105 paraffin-embedded tumors of breast cancer patients that we had previously characterized [13] (Table 1).

**Figure 3:**
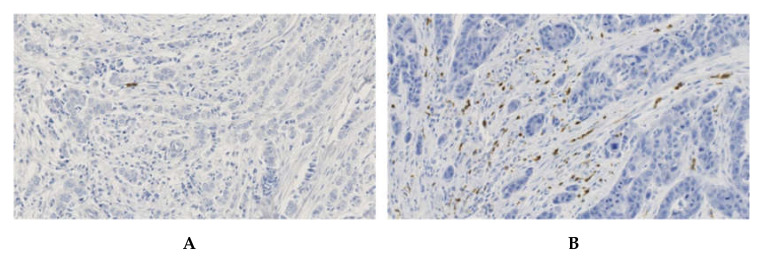
IHC patterns of CXCR2 staining in invasive breast cancers. (**A**) breast tumor with a low number of CXCR2-positive cells. (**B**) breast tumor with a high density of CXCR2-positive cells. Magnification 100×.

Variable density of CXCR2-positive cells was observed in invasive breast cancer samples (Figure 3). We confirmed in this large cohort of samples what we had observed during the IHC optimization process: scattered CXCR2-positive cells were restricted to the tumor stromal compartment and tumor cells were never immune-reactive.

**Figure 4:**
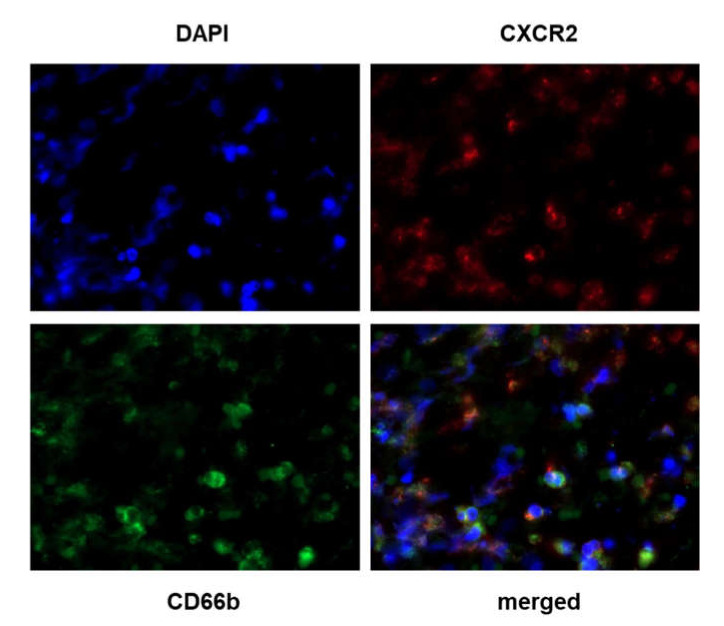
Colocalization of CXCR2 and CD66b staining. Double IF was performed using the CXCR2 rabbit polyclonal Ab HPA031999 combined with the CD66b mouse monoclonal Ab (clone 80H3) on samples showing various CXCR2 and CD66b expression levels following IHC. Briefly, the slides were incubated with a mix of DAPI (4′,6-diamidino-2-phenylindole) and secondary donkey anti-rabbit and anti-mouse Ab fluorescently labeled with Alexa Fluor 647 (CXCR2) and 488 (CD66b) dye molecules, respectively (ThermoFisher Scientific, Illkirch, France). Left upper quadrant: DAPI staining; right upper quadrant: CXCR2 staining; left lower quadrant: CD66b staining; right lower quadrant: merged image. Magnification 630×.

Stromal cells expressing CXCR2 had a granulocytic shape, suggesting that they could be neutrophils. In order to confirm this hypothesis, we performed immunofluorescence (IF) experiments. As IF requires the use of primary Ab from different species, we used the rabbit polyclonal HPA031999 Ab for the double CXCR2/CD66b IF procedure. By doing a dual labeling of CXCR2 and Cd66b on breast cancer tissues, we observed that most of the CXCR2 positive cells match with CD66b-positive neutrophils, confirming that neutrophils express high levels of CXCR2 as previously described (Figure 4) [18].

**Figure 5:**
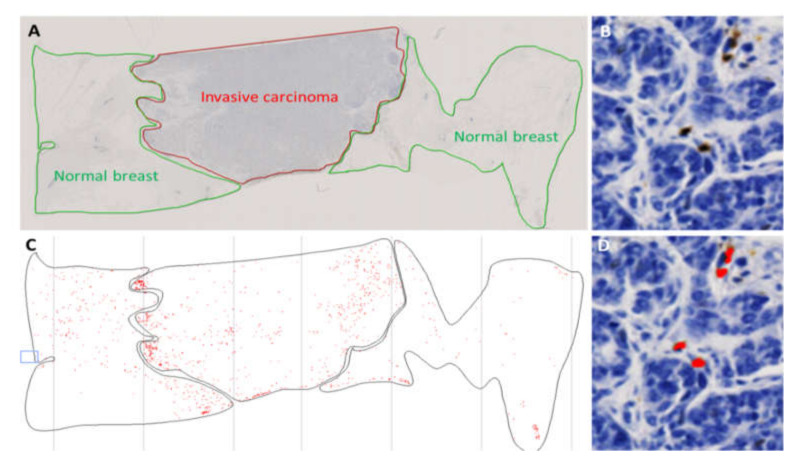
Quantitative assessment of immune-reactive cells in the tumor area. In (**A**), the invasive carcinoma area was identified as a region of interest (ROI, surrounded by a red line). Following manual thresholding for automatic detection of immune-reactive cells (**B**,**D**; Magnification 400×), the software identifies and quantifies positive cells within the ROI (red dots, (**C**,**D**)).

Quantification of IHC staining was performed by image analysis as described in the materials and methods and by delineating, in each sample, invasive carcinoma from the areas of in situ carcinoma, necrosis and normal breast tissue if any was present (Figure 5). All immunostaining assessments presented here were based on invasive carcinoma areas only.

We first compared levels of CXCR2, CD11b and CD66b expression in normal breast tissues from 21 patients undergoing plastic surgery to our cohort of 105 breast cancer patients. We observed that CXCR2 expression was significantly higher in breast cancer tissues compared to the normal one (*p* = 0.026), whereas CD11b expression was lower in cancer samples (*p* = 0.001) and CD66b similar in normal and cancer tissues (Table 2).

Focusing on breast tumors, we reported that CXCR2, CD11b or CD66b expression was not correlated to the age of patients, the histological type (ductal carcinoma versus others), the size of the tumors, or lymph node status (Table 3). On the other hand, CXCR2 (*p* = 0.002), CD11b (*p* = 0.032) and CD66b (*p* = 0.038) were positively correlated with high grade tumors (Table 3).

CXCR2 and CD11b were both correlated with estrogen receptor (ER)-negative tumors (*p* = 0.005 and *p* < 0.001, respectively), whereas CD66b was not (Table 3). Similarly, we observed a correlation of CXCR2 and CD11b with progesterone receptor (PR)-negative tumors (*p* = 0.002 and *p* < 0.001, respectively), but not for CD66b (Table 3). None of the three markers was significantly correlated with Her2 status (Table 3). When taking in account ER, PR and Her2 to differentiate TNBC (ER/PR− Her2−) from luminal hormone receptor-positive tumors (ER/PR+ Her2) or Her2-positive tumors (ER/PR± Her2+), it appeared that CXCR2, CD11b and CD66b expression was higher in triple-negative tumors compared to luminal and Her2-positive tumors (*p* < 0.001; *p* < 0.001 and *p* = 0.043, respectively; Table 3). No difference in the levels of the three markers could be seen between luminal and Her2-positive tumors (data not shown).

### 2.3. Analysis of the Correlation of CXCR2, CD11b and CD66b with Immune Infiltration of Tumors

Immune infiltration of tumors frequently involves multiple types of cells. It was thus interesting to determine if the neutrophils infiltration of breast tumors was correlated with T lymphocytes, B lymphocytes and macrophages. Levels of infiltration of T lymphocytes (CD3), B lymphocytes (CD20) and macrophages (CD68) were recovered from our previous analysis of the same cohort of patients [13]. By analyzing the possible correlation of CXCR2, CD11b and CD66b with these markers, we reported that high expression of CXCR2 and CD11b was correlated with a greater infiltration of T lymphocytes (*p* < 0.001 and *p* = 0.013, respectively) as well as of B lymphocytes (*p* = 0.007 and *p* = 0.003, respectively; Table 4). On the contrary, CD66b was not associated with T or B infiltration (Table 4). Interestingly, only CD11b staining (a marker of granulocytes including both neutrophils and macrophages) was correlated with macrophages infiltration (CD68 staining; *p* = 0.033), but CXCR2 and CD66b were not (Table 4).

### 2.4. CXCR2 Levels Are Correlated to AP-1 Levels

As NF-KB and AP-1 transcription factors are frequently involved in the regulation of inflammatory processes, we decided to evaluate whether CXCR2, CD11b and CD66b levels could be correlated to these factors. NF-KB and AP-1 expression had been quantified in our previous study based on the same cohort [13]. CXCR2 was correlated to AP-1 levels (*p* = 0.050), but not to NF-KB, while CD11b and CD66b were not correlated to any of these two factors (Table 4).

### 2.5. High CXCR2 Expression Is an Independent Prognostic Factor of Time to Relapse (TTR)

The median follow-up was 9.4 years (95% confidence interval (CI) (8.4–11.0)). Patients were divided in tertile groups of equal number of patients, according to their CXCR2 expression (low, medium and high). In univariate analysis, medium and high CXCR2 expression were associated with a lower risk of relapse (hazard ratio (HdR) of 0.231, 95% CI (0.073–0.731), *p* = 0.013 and 0.277, 95% CI (0.100–0.771), *p* = 0.014, respectively; Table 5). Of particular note, in univariate analysis, medium or high CD11b (HdR 1.318, 95% CI (0.457–3.803), *p* = 0.610 and 0.997, 95% CI (0.342–2.906), *p* = 0.995, respectively) and medium and high CD66b (HdR 1.626, 95% CI (0.584–4.529), *p* = 0.352 and 0.874, 95% CI (0.278–2.749), *p* = 0.818, respectively) were not significantly predictive of relapse.

**Figure 6:**
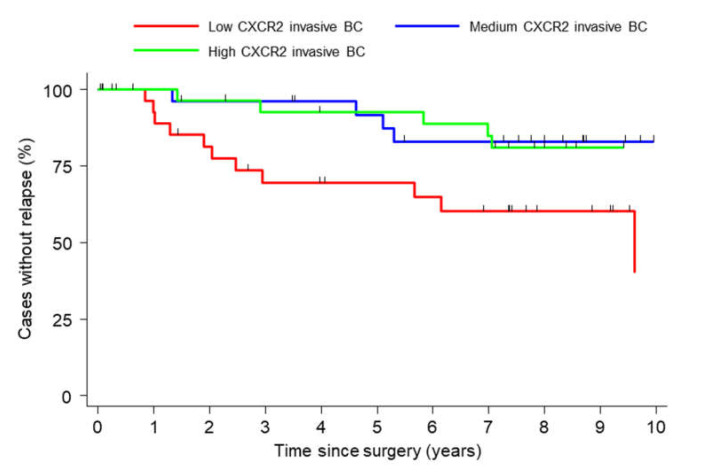
Time to relapse (TTR) according to CXCR2 expression.

In multivariate analysis, moderate or high levels of CXCR2 were independently associated with a lower risk of relapse (HdR 0.168, 95% CI (0.043–0.650), *p* = 0.010 and HdR 0.215, 95% CI (0.054–0.840), *p* = 0.028, respectively) as CD20 (HdR 0.252, 95% CI (0.068–0.932), *p* = 0.039), whereas AP-1 tended to be a negative prognostic factor (HdR 3.404, 95% CI (0.988–11.723), *p* = 0.052; Table 5). Kaplan–Meier curves showed that patients with low CXCR2 levels in the invasive tissue had a shorter time to relapse (TTR) compared to patients in the medium and high groups (5 years TTR: low CXCR2: 69.48% (48.05–83.45); medium: 91.78% (70.85–97.89) and high: 92.72% (73.89–98.13); Figure 6).

Patients were divided into three equal groups according to their levels of CXCR2 expression (low, medium and high) in invasive carcinoma and analyzed for TTR.

## 3. Discussion

In this study, we aimed to understand the potential involvement of the chemokine receptor CXCR2 in the breast tumor microenvironment. We and others have previously shown that human triple-negative breast cancers were expressing higher levels of most CXCR2 ligands (CXCL1, 2, 3, 5, 6, 7 and 8) than luminal or Her2-positive tumors [8,9,12,13,14,19]. This is presumably due to a coregulation of these chemokines, which are present in a very narrow region of chromosome 4 (4q21) [12]. Some of these chemokines such as CXCL1, CXCL5, CXCL6 and CXCL8 are produced at high levels by tumors cells, whereas some are also produced by blood cells, cancer associated fibroblasts or endothelial cells [12,14,16]. Based on the fact that aggressive TNBC tumors express high levels of CXCR2 ligands, it was important to study the expression of CXCR2 in breast tumors.

Using a cohort of breast cancer patients for which we had obtained data on multiple cytokines, including CXCL8 as well as immune infiltration, we have first performed an extensive review of available CXCR2 Ab, as CXCR2 immunostaining remains controversial. We selected seven Ab recommended for IHC by the manufacturers. Not surprisingly, the different Ab tested gave various types of staining. However, we managed to identify three different Ab that could provide a similar pattern of staining and which were also in agreement with ISH experiments to detect CXCR2 mRNA. These selected Ab and ISH were all showing CXCR2-positive cells only in the stromal compartment or within vessels but never on tumor cells. Moreover, the immune-reactive cells had a granulocytic shape, looking as possible neutrophils. We also observed in IF experiments of costaining with CXCR2 and the neutrophil marker CD66b that most of the CXCR2-positive cells were neutrophils. This is in agreement with earlier findings showing that neutrophils are expressing the highest levels of CXCR2 [20,21]. However, we cannot exclude that other types of stromal cells could express CXCR2. Answering this question will require a complete study by itself. CXCR2 might be also expressed at lower levels on endothelial cells, but this remains controversial [22,23] and was not objectified in our series. We also previously showed at the mRNA level that breast tumor cells did not express significant levels of CXCR2 mRNA [12]. Moreover, to confirm the specificity of the CXCR2 Ab (clone E-2) that we selected to perform the complete study, we tested this Ab on HEK-293 cells transfected with CXCR2 cDNA, further reinforcing that this Ab was adequate.

The pattern of CXCR2 expression in breast cancer remains a controversial question, presumably due to the use of a variety of Ab, with distinct specificities. A previous small study has shown that another CXCR2 Ab (no more commercially available) stained tumor cells in a series of only 37 patients [24], in which no validation of the Ab used was presented. In the same line, Xu et al. [25] used an Ab from Sigma Aldrich (c-6348), which stained also breast tumor cells. However, this Ab is not intended for use in IHC experiments but for flow cytometry and neutralizing studies. Thus, we did not select this Ab to be tested in the present study. Moreover, this study never shows any specificity controls such as positive or negative controls of IHC. In addition, IF experiments presented in the study from Xu et al. shows a clear cytoplasmic staining, which is in disagreement with the fact that CXCR2 is a transmembrane receptor. So, together, these data could explain the difference in terms of results with our study. Finally, a third study from Romero-Moreno et al. [26] used another Ab (ab14935) mainly to stain mouse tissue. They report also a staining of bone and marrow cells from a human femoral head, but the nature of the cells expressing CXCR2 was not precise. One could speculate that it could be neutrophils as these cells are highly present among marrow cells.

We next compared the expression of CXCR2, CD11b and CD66b between normal breast tissue and breast cancers. We observed that CXCR2 was more expressed in breast tumors than in a normal breast, whereas CD11b was present at lower levels in breast tumors and CD66b was not different between normal and cancer tissues. Interestingly, if CXCR2 and CD66b should label the neutrophils, our results suggest that CXCR2 is an independent marker of neutrophils, with some neutrophils that do not express CXCR2. Higher expression of CXCR2 in tumor tissues compared to normal tissue has also been reported in the pancreas, lung or colon cancers [27,28,29]. It is also interesting to note that CXCR4, another chemokine receptor, shows a higher expression in breast cancer tissue, whereas its ligand CXCL12 is downregulated in the same tissue, which is in contrast to the situation observed for CXCR2 [30].

When looking in more details to breast cancer tissues, we show that CXCR2, CD11b and CD66b high levels were correlated to a high tumor grade and to TNBC tumors. To our knowledge, CD11b and CD66b have not been evaluated so far in primary breast tumors in terms of association with clinical parameters. It is interesting to note that CXCR2 expression parallels one of its ligands, which is also expressed at higher levels in TNBC tumors [12,13,14], even if we did not observe a strict correlation between CXCL8 and CXCR2 in this study (data not shown). Interestingly, other chemokines or their receptors can be associated with ER. This is the case of CXCL12, which its expression is regulated by estrogens but can also activate ER activity [31]. Moreover, CXCR4, one of the receptor of CXCL12, is a poor prognosis factor in breast cancer patients, independently of ER status, and its high expression has been shown to promote estrogen-independent tumor growth in vivo [32].

We also reported that high staining for CD11b and CXCR2, but not CD66b were correlated to a higher infiltration of B and T lymphocytes. Whether this is a consequence of the mutual attraction of each type of cells, or that all these cells are infiltrating the tumor because of the presence of common chemoattractants remains to be addressed. We have previously reported that breast tumors with a high content of CCL2 or CCL4 had a higher infiltration of B and T lymphocytes [13]. Moreover, one hypothesis for the increase in CXCR2 positive cells could be the higher release of CXCR2 ligands by other cells. In particular, we and others have shown that both CXCL1 and CXCL8, ligands of CXCR2, could be produced at higher levels by mesenchymal stem cells or fibroblasts, which have been “educated” by TNBC cells [16,33,34]. Similar overexpression of CXCR2 ligands occurs also for breast cancer cells and could represent another source of chemokines production [9,12,13,14,19].

In the search of potential molecular mechanisms accounting for a high expression of CXCR2, we were able to show that it was correlated to high expression of AP-1 transcription factors but not NF-KB. This is reminiscent to CXCL8, one of the ligand of CXCR2, which its expression is also correlated to AP-1 activity [12,13].

We also investigated the potential of CXCR2, CD11b and CD66b as prognostic factors. We showed in a univariate and multivariate analysis that high CXCR2 expression was associated with a better prognosis in terms of TTR, whereas CD11b and CD66b were not. This further reinforces our data showing that CXCR2 and CD66b are not equivalent markers of neutrophils. This could suggest potentially different functions of CXCR2 negative and positive neutrophils. In line with this, it was shown on neutrophils isolated from the blood of patients that the percentage of CXCR2 positive neutrophils was lower in breast cancer patients compared to healthy patients [35]. Another study has also shown that CD66b was an independent poor prognosis factor for disease-free survival of breast cancer patients. CD66b marker, as a marker of neutrophils will require additional studies to decide if it can be used as a prognosis factor. The survival analysis was performed on the whole cohort of breast cancer patients. Due to the number of patients included in the cohort, it was not possible to analyze TTR for each group and in particular TNBC separately, as it would not have provided enough statistical power. However, for the group ER+/PR+ patients, which was large enough to be analyzed, we observed the same favorable TTR with high CXCR2 patients (data not shown). It would be interesting in the future to determine the prognostic value of CXCR2 for each subgroup of breast cancer patients. In a variety of cancers, it has been shown that CXCR2 was expressed in cancer cells and frequently associated with a poor prognosis [9,36]. It is interesting to mention that the prognosis value of CXCR2 has remained so far poorly studied in breast cancer, whereas it has been analyzed more thoroughly in other cancers. Xu et al. have shown that a high level of CXCR2 was a poor prognosis factor of disease free survival in breast cancer [25]. This is in disagreement with our results, but could be explained by the fact the Ab that they used stained mainly cancer cells and not stromal cells, as mentioned earlier. Interestingly, CXCR2 could be also a potential cancer stem-like cell marker, as a costaining of some breast cancer cells with NANOG and SOX2 has been reported and also that CXCR1/2 inhibition decreases mammosphere formation [24,37]. This is reminiscent of the role of another chemokine receptor, CXCR1, which binds some ELR chemokines and is critical for the functionality of cancer stem cells [38]. Overall, the discrepancy observed for the prognosis value of CXCR2 in breast cancer might be related to the lack of specificity of the Ab used. As we conducted various validation tests to insure the specificity of CXCR2 staining reported here, we believe that CXCR2 is not expressed in breast epithelial cells. Staining of cancer cells would definitely alter the value of CXCR2 for outcome. Altogether, our results further reinforce the unique role of stromal compartment in cancer development.

## 4. Materials and Methods

### 4.1. Patients and Tumor Samples

One hundred and five formalin-fixed paraffin-embedded tumors samples, corresponding to the frozen samples previously used in our first study [13], were selected from the Montpellier Cancer Institute (ICM) biological resources center for IHC analyses. The tumors were graded according to the modified Nottingham SBR system and categorized according to the sixth edition of the AJCC (American Joint Committee on Cancer) Cancer Staging Manual for pTNM staging. The ER, PR and Her2 status were determined at the protein level by immunohistochemistry. When equivocal, Her2 results were confirmed by fluorescence ISH. The same samples as those previously used in our first study were selected for IHC analyses [13], as previously published. A follow up was updated on September 2018 by review of the medical files. The clinical and pathological characteristics of the patients as well the measure of B lymphocytes, T lymphocytes, macrophages infiltration and the levels of AP-1 and NF-KB transcription factors are described in Table 1.

### 4.2. Ethics Approval and Consent to Participate

Breast tumor surgical specimens were provided by the biological resource center (Biobank number BB-0033-00059) after approval of the Montpellier Cancer Institute Institutional Review Board (ICM-CORT-2018-06, 11 March 2018), following the Ethics and Legal national French dispositions for the patients’ information and consent. All patients were informed before surgery that their surgical specimens might be used for research purposes.

### 4.3. Immunohistochemistry

IHC analyses were performed using 3-μm-thin sections from formalin-fixed paraffin-embedded tissue blocks on the Dako Autostainer Link 48 platform (Dako-Agilent, Glostrup, Denmark). Ab clones, suppliers, antigen retrieval procedures, dilutions and staining protocols are listed in Table S1. The PTLink system (Dako-Agilent) was used for pretreatment, allowing simultaneous deparaffinization, rehydration and antigen retrieval. Heat-induced antigen retrieval was executed for 15 min at 95 °C in high (pH 9) or low (pH 6.1) pH buffer (Dako-Agilent) according to the Ab. Endogenous peroxidase was quenched using Flex Peroxidase Block (Dako-Agilent) for 5 min at room temperature. Slides were then incubated with the selected Ab (anti-CXCR2 mouse monoclonal Ab Sc-7304 at 1/500, Santa Cruz Technology, Dallas, TX, USA; anti-CD11b rabbit monoclonal Ab EP45 at 1/400, BioSB, Santa Barbara, CA, USA or anti-CD66b mouse monoclonal Ab 80H3 at 1/200, BioRad, Marnes-la-Coquette, France) for 30 min at room temperature. After 2 rinses in buffer, the slides were incubated with a horseradish peroxidase-labeled polymer coupled to secondary anti-mouse and anti-rabbit Ab for 20 min, followed by appliance of 3,3′-diaminobenzidine for 10 min as a substrate. Counterstaining was performed using Flex Hematoxylin (Dako-Agilent) followed by washing the slides under tap water for 5 min. Finally, slides were mounted with a coverslip after dehydration. A set of samples were also processed with irrelevant IgG isotypic control at the same concentration as CXCR2 Ab, and used as negative control.

The NanoZoomer slide scanner system (Hamamatsu Photonics, Massy, France) was used to digitalize glass slides at the 20× objective. The invasive component of each sample was manually surrounded on the virtual slide by a pathologist and identified as a region of interest (ROI). Necrotic areas, in situ carcinoma and normal breast were excluded from the ROI. Following the calibration of size, shape and color parameters, immune-reactive cells were automatically quantified with the HistoLab^®^ Image Analysis Software (Microvision, Evry, France) as previously described [39]. Density of immune-reactive cells in the ROI (Figure 5), recorded as the number of positive cells per cm^2^ of tumor surface, was finally matched to clinicopathological data.

### 4.4. In Situ Hybridization

ISH was performed using specific HsCXCR2 probes and an RNAscope^®^ 2.5 HD detection kit (Bio-Techne, Abingdon, UK) according to the manufacturer’s instruction. Serial sections were used for CXCR2 IHC and ISH procedures. Probes that target the housekeeping *Homo sapiens* PPIB gene and the bacterial DapB gene were used as a positive and negative control, respectively.

### 4.5. Immunofluorescence

Double IF was performed using the CXCR2 rabbit polyclonal Ab HPA031999 combined with the CD66b mouse monoclonal Ab (clone 80H3) on three selected samples showing various CXCR2 and CD66b expression levels following IHC. Briefly, 3-μm-thin sections were submitted to the antigen retrieval procedure using the low pH buffer (Dako-Agilent). Sections were incubated with donkey serum for 30 min at room temperature to block non-specific binding of immunoglobulins and then, with a combination of CXCR2 (1/100) and CD66b (1/200) for 16 h at +4 °C. After washing with PBS Tween, the slides were incubated with a mix of DAPI and secondary donkey anti-rabbit and anti-mouse Ab fluorescently labeled with Alexa Fluor 647 (CXCR2) and 488 (CD66b) dye molecules, respectively (ThermoFisher Scientific, Illkirch, France). The slides were mounted with Mowiol (Sigma-Aldrich, Darmstadt, Germany) for fluorescence microscopy.

### 4.6. Transfection of Human CXCR2 in HEK-293 Cells

HEK-293 cells were plated in 10 cm dishes and transfected using JetPEI (Ozyme, St Quentin Yvelines, France) according to the manufacturer’s recommendations, using 10 µg of pcDNA3 (control vector) or cDNA3-hCXCR2 expression vector (Interchim, Montluçon, France). After 18 h incubation, the medium was removed and the cells were placed into a fresh medium. Twenty-four hours later, cells were harvested and either included as a pellet for IHC experiments or prepared for protein extraction.

### 4.7. Protein Extraction and Western Blot

For protein extraction, HEK-293 cells transfected or not with human CXCR2 cells were lyzed in R1 buffer (20 mM Hepes pH 7.3; 20 mM MgCl2, 150 mM NaCl, 0.75% NP-40), supplemented with protease inhibitor cocktail (Roche, Meylan, France). Fifteen micrograms of total protein extracts were separated by SDS-PAGE and subjected to Western blot analyses using CXCR2 Ab (Santa Cruz sc-7304, 1/1000). Immunoreactivity was detected with the Merck Millipore ECL system (Molsheim, France).

### 4.8. Statistics

Patients’ characteristics were summarized using descriptive statistics. Continuous variables were described using medians and ranges, and categorical variables with frequencies and percentages.

Association between biomarkers and tumor characteristics was tested using the Kruskal–Wallis test. A *p* value of less than 0.05 was considered statistically significant.

TTR was defined by the interval (calculated in years) between the date of surgery and the date of the first event between local and metastatic recurrence. Only untreated primary tumors were selected for survival analysis (*N* = 90). TTR was estimated using the Kaplan–Meier method, and median follow-up durations using the reverse Kaplan–Meier method. Survival curves with their log-rank tests were generated. Censored data were descriptively summarized for the different equal groups.

Variables with a *p*-value less than 0.25 from univariate analysis, including prognostic factors, were introduced in the multivariate model with a set of forced variables (age, SBR grade and immune type). Then, backward and stepwise selection strategies of the non-forced variables were performed. The selection procedure consists of removing variables having the largest *p*-value greater than 0.10 from the model.

The multivariate analysis was performed using a Cox model adjusted for prognostic factors of survival and potential cofounders. HdRs are presented on a descriptive basis with 95% CI. Statistical analyses were performed using STATA 13.0 (StataCorp, College Station, TX, USA).

## 5. Conclusions

In conclusion, our results showed that stromal CXCR2 levels were correlated with high grade breast tumors and TNBC phenotype as previously reported for its ligands. However, in the meantime, high CXCR2 levels were also associated with a lower risk of relapse. This study reinforces the potential role of tumor microenvironment in patient outcome.

## Figures and Tables

**Table 1:** Characteristics of the studied population (*N* = 105).

Variables	*N*	%
**Age**		
≤50 years	36	34.3
50–65 years	31	29.5
>65 years	38	36.2
**Type**		
Ductal carcinoma	77	73.3
Others	28	26.7
**SBR Grade**		
I/II	73	69.5
III	32	30.5
**pT**		
pT1	42	40.4
pT2/pT3/pT4	62	59.6
Missing	1	/
**pN**		
pN0	50	50
pN1/pN2/pN3	50	50
Missing	5	/
**pM**		
pM0	80	76.2
pM1	1	1
pMX	24	22.9
**ER**		
Negative	36	34.3
Positive	69	65.7
**PR**		
Negative	52	49.5
Positive	53	50.5
**HR (if ER+ and/or PR+)**		
Negative	29	27.6
Positive	76	72.4
**Her2**		
Negative	94	89.5
Positive	11	10.5
**Immunophenotype**		
ER/PR− Her2−	23	21.9
ER/PR± Her2+	11	10.5
ER/PR+ Her2−	71	67.6
**CD3**		
Negative	54	51.4
Positive	51	48.6
**CD20**		
Negative	74	70.5
Positive	31	29.5
**CD68**		
Negative	5	4.8
Positive	100	95.2
**AP-1**		
Negative	34	33
Positive	69	67
Missing	2	/
**NF-KB**		
Negative	29	28.2
Positive	74	71.8
Missing	2	/

Abbreviations: ER, estrogen receptor; HR, Hormone Receptors; PR, progesterone receptor; SBR, Scarff–Bloom–Richardson.

**Table 2:** Expression of CXCR2, CD11b and CD66b in normal and cancer breast tissues.

Variables	CXCR2			CD11b			CD66b		
	*N*	Median (Range)	*p*-Value	*N*	Median (Range)	*p*-Value	*N*	Median (Range)	*p*-Value
**Breast**									
Normal	21	145.83 (24.20:714.60)	0.026	21	242.90 (48.96:1014.74)	0.001	21	85.46 (25.43:375.67)	0.587
Cancer	95	269.07 (19.38:4539.93)		100	97.60 (0.71:85,867.80)		98	68.96 (0.00:1920.86)	

Values are expressed as median (range) of density of positive cells/cm^2^; *p*-value determined using a non parametric Kruskal–Wallis test.

**Table 3:** Correlations of CXCR2, CD11b and CD66b expression with clinicopathological features.

Variables	CXCR2			CD11b			CD66b		
	*N*	Median (Range)	*p*-Value	*N*	Median (Range)	*p*-Value	*N*	Median (Range)	*p*-Value
**Age**									
≤50 years	35	344.81 (31.4:7789.8)	0.546	35	125.65 (4.7:13376.5)	0.142	35	48.37 (4.4:10032.0)	0.345
50–65 years	31	268.28 (40.6:5085.6)		31	79.02 (2.8:2963.4)		30	52.17 (0.0:4842.6)	
>65 years	34	406.60 (38.6:2201.2)		36	146.6 (12.2:1118.5)		37	90.0 (4.3:1501.9)	
**Type**									
Ductal carcinoma	73	351.60 (31.45:7789.79)	0.468	75	146.54 (2.81:13376.50)	0.357	76	70.36 (0.00:4842.61)	0.355
Others	27	295.24 (38.59:4998.79)		27	79.02 (11.99:2579.57)		26	35.29 (0.00:10031.97)	
**SBR Grade**									
I/II	69	298.45 (31.45:5085.62)	0.002	71	111.87 (2.81:4568.04)	0.032	71	36.08 (0.00:4842.61)	0.038
III	31	698.38 (55.23:7789.79)		31	239.05 (9.36:13376.50)		31	89.98 (5.45:10031.97)	
**pT**									
pT1	41	263.28 (31.45:4998.79)	0.102	42	90.11 (4.69:5192.38)	0.051	42	48.56 (0.00:10031.97)	0.119
pT2/pT3/pT4	58	379.24 (40.61:7789.79)		59	200.09 (2.81:13376.50)		59	85.58 (0.00:4842.61)	
**pN**									
pN0	49	384.97 (31.45:7789.79)	0.561	49	151.44 (4.69:13376.50)	0.391	48	50.24 (4.41:10031.97)	0.971
pN1/pN2/pN3	46	320.31 (38.59:3706.72)		48	125.30 (2.81:9547.40)		49	48.37 (0.00:1501.86)	
**ER**									
Negative	34	540.59 (83.49:7789.79)	0.005	35	373.09 (11.99:13376.50)	<0.001	35	79.85 (0.00:10031.97)	0.065
Positive	66	296.85 (31.45:5085.62)		67	85.91 (2.81:4568.04)		67	34.50 (0.00:4842.61)	
**PR**									
Negative	51	472.48 (38.59:7789.79)	0.002	51	262.77 (11.99:13376.50)	<0.001	51	75.46 (0.00:10031.97)	0.08
Positive	49	250.94 (31.45:2025.37)		51	78.25 (2.81:4568.04)		51	32.98 (0.00:1501.86)	
**Her2**									
Negative	90	343.64 (31.45:7789.79)	0.37	92	146.08 (2.81:13376.50)	0.84	92	53.00 (0.00:10031.97)	0.653
Positive	10	285.67 (141.87:1067.14)		10	103.49 (16.43:515.78)		10	52.12 (5.45:570.98)	
**Immunophenotype**									
ER/PR− Her2−	23	1082.20 (154.6:7789.8)	<0.001	23	555.08 (12.0:13376.5)	<0.001	23	108.13 (6.4:10032.0)	0.043
ER/PR± Her2+	10	285.67 (141.9:1067.1)		10	103.49 (16.4:515.8)		10	52.12 (5.5:571.0)	
ER/PR+ Her2−	67	295.2 (31.4:5085.6)		69	85.9 (2.8:4568.0)		69	34.5 (0.0:4842.6)	

Values are expressed as median (range) of density of positive cells/cm^2^; *p*-value determined using a non parametric Kruskal–Wallis test.

**Table 4:** Correlations of CXCR2, CD11b and CD66b expression with immune infiltration and pathways.

Variables	CXCR2			CD11b			CD66b		
	*N*	Median (Range)	*p*-Value	*N*	Median (Range)	*p*-Value	*N*	Median (Range)	*p*-Value
**CD3**									
Negative	52	253.21 (31.45:5085.62)		53	85.91 (2.81:5192.38)	0.013	52	48.44 (0.00:4842.61)	0.828
Positive	48	490.63 (55.23:7789.79)	<0.001	49	205.85 (14.95:13376.50)		50	60.56 (0.00:10031.97)	
**CD20**									
Negative	70	292.12 (31.45:7789.79)	0.007	71	98.49 (2.81:13376.50)	0.003	71	48.37 (0.00:4842.61)	0.608
Positive	30	481.76 (156.36:4998.79)		31	291.71 (19.02:9547.40)		31	75.46 (6.41:10031.97)	
**CD68**									
Negative	5	151.78 (31.45:725.11)	0.053	5	41.97 (4.69:151.44)	0.033	4	19.96 (4.41:159.43)	0.196
Positive	95	344.81 (38.59:7789.79)		97	146.54 (2.81:13376.50)		98	55.73 (0.00:10031.97)	
**AP-1**									
Negative	31	268.59 (38.59:2201.23)	0.05	32	88.07 (9.36:4568.04)	0.079	32	32.40 (4.34:1416.34)	0.311
Positive	67	384.97 (31.45:7789.79)		68	178.18 (2.81:13376.50)		68	77.65 (0.00:10031.97)	
**NF-kB**									
Negative	29	288.99 (55.23:5085.62)	0.516	29	124.96 (9.36:9547.40)	0.64	28	42.22 (0.00:10031.97)	0.857
Positive	69	362.21 (31.45:7789.79)		71	146.54 (2.81:13376.50)		72	53.00 (0.00:2748.86)	

Values are expressed as median (range); *p*-value determined using a non parametric Kruskal–Wallis test.

**Table 5:** Time to relapse univariate and multivariate analyses.

Parameter	Univariate Analysis		Multivariate Analysis	
	HdR (95% CI)	*p*-Value	HdR (95% CI)	*p*-Value
**SBR Grade**				
I/II	1.000		1.000	
III	1.117 (0.465–2.685)	0.805	1.143 (0.382–3.420)	0.811
**Immune Type**				
ER/PR− Her2−	1.000		1.000	
ER/PR± Her2+	2.629 (0.749–9.233)	0.131	1.718 (0.311–9.498)	0.535
ER/PR+ Her2−	1.038 (0.358–3.006)	0.945	0.553 (0.137–2.237)	0.406
**Age at Diagnosis**				
<50	1.000		1.000	
50–65	0.835 (0.317–2.200)	0.714	0.641 (0.215–1.910)	0.425
>65	0.682 (0.230–2.020)	0.490	0.537 (0.161–1.789)	0.311
**CD20**				
Negative	1.000		1.000	
Positive	0.356 (0.119–1.065)	0.065	0.252 (0.068–0.932)	0.039
**AP-1**				
Negative	1.000		1.000	
Positive	2.270 (0.761–6.773)	0.142	3.404 (0.988–11.723)	0.052
**pT**				
T1	1.000		1.000	
T2/T3/T4	1.727 (0.704–4.238)	0.233	2.486 (0.909–6.797)	0.076
**CXCR2 invasive**				
low	1.000		1.000	
medium	0.231 (0.073–0.731)	0.013	0.168 (0.043–0.650)	0.010
high	0.277 (0.100–0.771)	0.014	0.215 (0.054–0.840)	0.028
**CD11b invasive**				
low	1.000			
medium	1.318 (0.457–3.803)	0.610		
high	0.997 (0.342–2.906)	0.995		
**CD66b invasive**				
low	1.000			
medium	1.626 (0.584–4.529)	0.352		
high	0.874 (0.278–2.749)	0.818		

*p*-value determined using a Cox proportional-hazards model.

## Supplementary Materials

The following are available online at https://www.mdpi.com/2072-6694/12/8/2076/s1, Figure S1: Representative examples of CXCR2 staining in formalin-fixed paraffin-embedded samples, Figure S2: Validation of the CXCR2 immuno-staining in invasive breast carcinoma, Figure S3: Whole Western Blot for Figure 2A, Table S1: Immuno-histochemical reagents and protocols.

## Abbreviations

Ab	antibody
CI	Confidence interval
ER	Estrogen Receptor
Her2	Human Epidermal Growth Factor Receptor-2
HR	hormone receptor
HdR	hazard ratio
IF	immunofluorescence
IHC	Immunohistochemistry
ISH	in situ hybridization
PR	Progesterone Receptor
ROI	Region of interest
SBR	Scarff–Bloom–Richardson
TNBC	Triple-Negative Breast Cancer
TTR	Time To Relapse
