# Supplementary material for: Prognostic Value of CXCR2 in Breast Cancer
Source: Cancers (Basel). 2020 Jul 27;12(8):2076. doi: 10.3390/cancers12082076 (PMC7465124; doi:10.3390/cancers12082076)
Supplement: Supplementary file 1 [file cancers-12-02076-s001.pdf]

cancers

Article

Prognostic Value of CXCR2 in Breast Cancer

Florence Boissière-Michot, William Jacot, Julien Fraisse, Sophie Gourgou, Colin Timaxian and Gwendal Lazennec

Supplementary Materials

Table S1. Immuno-histochemical reagents and protocols.

Target	Type	Clone	Source Reference	Antigen Retrieval	Dilution	Staining Protocol	IHC Validation
CXCR2	Rabbit polyclonal	-	LSBio LS-A804	-	-	-	No
CXCR2	Mouse monoclonal	5E8-C7-F10	Affimetrix #12-1829	-	-	-	No
CXCR2	Mouse monoclonal	48-311	R&D systems MAB331	-	-	-	No
CXCR2	Mouse monoclonal	6D499	Acris AM08392	-	-	-	No
CXCR2	Mouse monoclonal	19	InVitrogen AHR1532	pH 9	1/500	Linker mouse Envision™ Flex+	Yes
CXCR2	Mouse monoclonal	E-2	Santa Cruz Sc-7304	pH 9	1/500	Linker mouse Envision™ Flex+	Yes
CXCR2	Rabbit polyclonal	-	Atlas Ab HPA03199	pH 6.1	1/100	Linker rabbit Envision™ Flex+	Yes
CD11b	Rabbit monoclonal	EP45	BioSB BSB 6440	pH 9	1/400	Linker rabbit Envision™ Flex+	Yes
CD66b	Mouse monoclonal	80H3	Bio-Rad MCA216	pH 6.1	1/200	Envision™ Flex	Yes

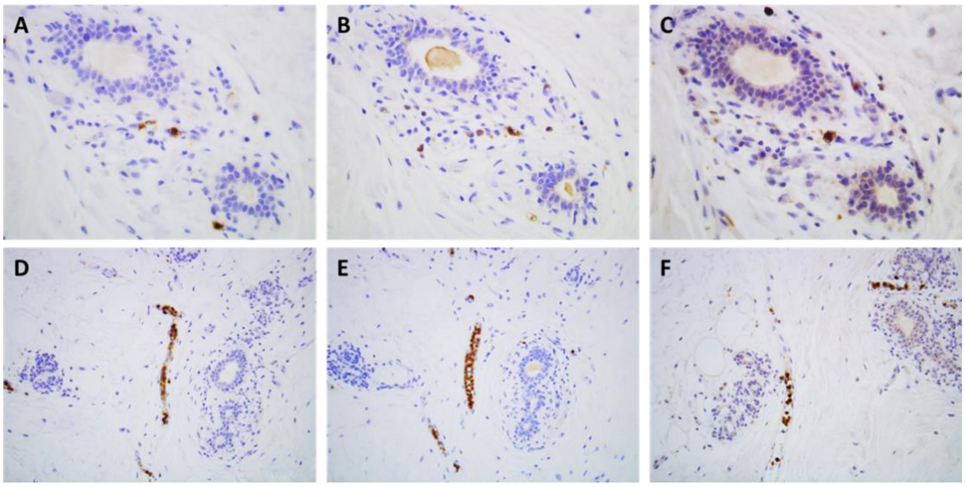

Figure S1. Representative examples of CXCR2 staining in formalin-fixed paraffin-embedded samples. Immunostaining of CXCR2 in serial sections of human breast samples using 3 Ab (**A** and **D**: clone E-2; **B** and **E**: rabbit polyclonal Ab HPA03199; **C** and **F**: clone 19). Note a consistent pattern between the 3 Ab with staining of scattered cells within the stroma (**A**,**B**,**C**) and inside blood vessels (**D**,**E**,**F**), and absence of staining of epithelial cells. Immuno-peroxidase 400× (**A**,**B**,**C**), 200× (**D**,**E**,**F**).

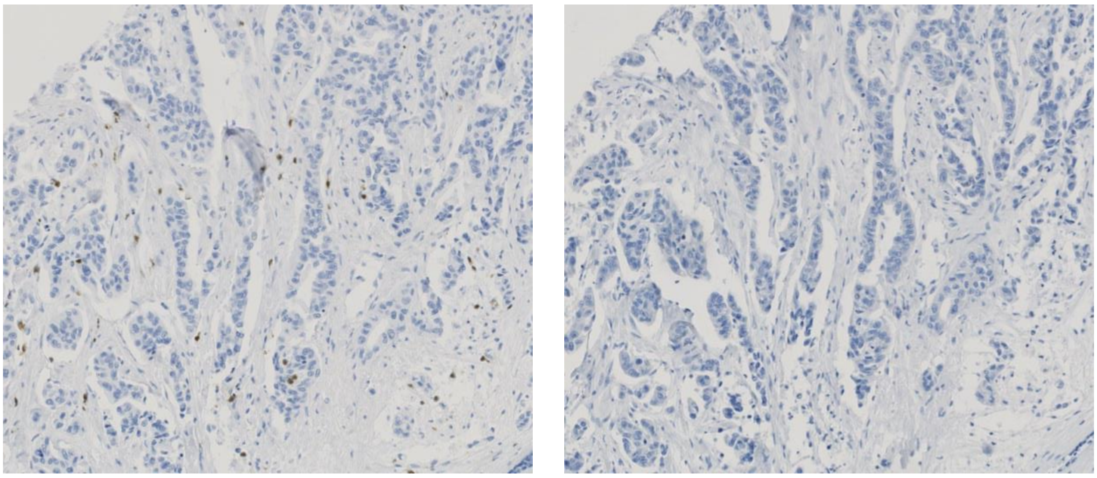

A B

Figure S2. Validation of the CXCR2 immuno-staining in invasive breast carcinoma. (**A**) One section was labelled with the E-2 mouse monoclonal IgG1 anti-CXCR2 Ab at 0.4 µg/mL. (**B**) Serial section was processed as the section shown in A, but with irrelevant IgG1 used at the same concentration. Immuno-peroxidase 200×.

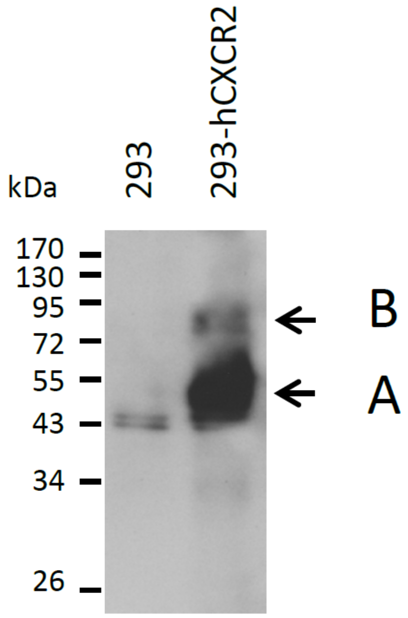

	bands Densitometry
control A	19.43
CXCR2 A	110.78
control B	3.92
CXCR2 B	61.00

ratio CXCR2/ control	
Band A	5.70
Band B	15.55

Figure S3. Whole Western Blot for figure 2A.
